# Crossmodal Connections of Primary Sensory Cortices Largely Vanish During Normal Aging

**DOI:** 10.3389/fnagi.2018.00052

**Published:** 2018-03-02

**Authors:** Julia U. Henschke, Frank W. Ohl, Eike Budinger

**Affiliations:** ^1^Department Systems Physiology of Learning, Leibniz Institute for Neurobiology, Magdeburg, Germany; ^2^Department Genetics, Leibniz Institute for Neurobiology, Magdeburg, Germany; ^3^German Center for Neurodegenerative Diseases within the Helmholtz Association, Magdeburg, Germany; ^4^Institute of Cognitive Neurology and Dementia Research (IKND), Otto-von-Guericke-University Magdeburg, Magdeburg, Germany; ^5^Center for Behavioral Brain Sciences, Magdeburg, Germany; ^6^Institute of Biology, Otto-von-Guericke-University Magdeburg, Magdeburg, Germany

**Keywords:** anatomy, cortex, multisensory, rodent, thalamus, tract-tracing

## Abstract

During aging, human response times (RTs) to unisensory and crossmodal stimuli decrease. However, the elderly benefit more from crossmodal stimulus representations than younger people. The underlying short-latency multisensory integration process is mediated by direct crossmodal connections at the level of primary sensory cortices. We investigate the age-related changes of these connections using a rodent model (Mongolian gerbil), retrograde tracer injections into the primary auditory (A1), somatosensory (S1), and visual cortex (V1), and immunohistochemistry for markers of apoptosis (Caspase-3), axonal plasticity (Growth associated protein 43, GAP 43), and a calcium-binding protein (Parvalbumin, PV). In adult animals, primary sensory cortices receive a substantial number of direct thalamic inputs from nuclei of their matched, but also from nuclei of non-matched sensory modalities. There are also direct intracortical connections among primary sensory cortices and connections with secondary sensory cortices of other modalities. In very old animals, the crossmodal connections strongly decrease in number or vanish entirely. This is likely due to a retraction of the projection neuron axonal branches rather than ongoing programmed cell death. The loss of crossmodal connections is also accompanied by changes in anatomical correlates of inhibition and excitation in the sensory thalamus and cortex. Together, the loss and restructuring of crossmodal connections during aging suggest a shift of multisensory processing from primary cortices towards other sensory brain areas in elderly individuals.

## Introduction

Multisensory integration recruits higher-level association cortex, but also low-level and even primary sensory cortices like the primary auditory (A1), somatosensory (S1), and visual cortex (V1). Neurons in these brain regions respond to their own (“matched”) sensory modality but also to other (“non-matched”) modalities and they receive convergent anatomical inputs from multiple senses (for review, e.g., Schroeder and Foxe, 2005; Driver and Noesselt, 2008; Stein and Stanford, 2008; Budinger and Scheich, 2009; Murray et al., 2016; Meredith and Lomber, 2017). The underlying anatomical pathways of these crossmodal inputs include a thalamocortical (from thalamus to cortex) and a corticocortical (intracortical) system, which may both function to preferentially serve short-latency integration processes in the primary sensory areas (Budinger and Scheich, 2009; Sperdin et al., 2009; Henschke et al., 2015). Functionally, combining information across the sensory modalities improves sensory performance of individuals (crossmodal facilitation effect; Welsh and Warren, 1986; Stein and Meredith, 1993), for example, by decreasing response times (RTs; e.g., humans: Gielen et al., 1983; Teder-Sälejärvi et al., 2002; Noesselt et al., 2010; animals: Sakata et al., 2004; Gleiss and Kayser, 2012).

Psychophysical studies have shown that older adults typically have longer RTs to unimodal and crossmodal stimuli but their RT benefit for crossmodal vs. unimodal stimuli is larger than for younger adults (for review, e.g., Mozolic et al., 2012; Freiherr et al., 2013; de Dieuleveult et al., 2017). This means that the RT gain to crossmodal stimuli of older individuals is more larger than those of younger individuals (Laurienti et al., 2006). One study even demonstrated faster RTs of older adults to audiovisual stimuli when compared to young adults (Peiffer et al., 2007). However, very little is known about the anatomy underlying these differences in multisensory performance (i.e., longer RTs but greater RT benefit) between the young and elderly. Currently, three different, but not mutually exclusive, mechanisms have been suggested (for review, e.g., Mozolic et al., 2012; Freiherr et al., 2013; de Dieuleveult et al., 2017):

(i) An age-related deterioration of the individual sensory systems, mainly by a loss or reduced function of auditory, tactile, and visual receptor cells (for review, e.g., Liu and Yan, 2007; Shaffer and Harrison, 2007; Owsley, 2011), may reduce the available unisensory and thus also multisensory information.
(ii) An age-related degeneration of myelin sheaths surrounding nerve fibers (for review, e.g., Peters, 2009), paralleled by an overall reduction of brain volume (for review, e.g., Hedman et al., 2012), may cause slower neuronal conductance (e.g., Waxman, 1980; Madden et al., 2004) and thus longer uni- and multisensory RTs.
(iii) Age-related changes in brain recruitment strategies, such as the increasing shift of activity from posterior (more sensory) to anterior (more cognitive) brain regions and between hemispheres (for review, e.g., Davis et al., 2008; Grady, 2012), involve different neuronal pathways and consequently lead to different uni- and multisensory RTs and performance.

However, until now, there have been no studies investigating age-related differences of the actual anatomical connectivity underlying multisensory integration processes between young and old. Thus, we examined potential alterations of modality-specific (sensory matched) and crossmodal (sensory non-matched) anatomical connections of early multisensory processing stages, namely the primary sensory cortices, during aging. We quantified the sensory thalamic and cortical inputs into the primary sensory cortices A1, S1, and V1 in both adults (P120) and elderly (P1000) animals using a well-suited rodent model species (Mongolian gerbil). To do this, we used a retrograde tract tracing method (Fluorogold, FG) and, subsequently, investigated possibly underlying cellular mechanisms using immunohistochemistry for markers of cell apoptosis (Cysteinyl-aspartate Specific Protease 3, CASP3), axonal plasticity (Growth Associated Protein 43, GAP43), and a calcium-binding protein (Parvalbumin, PV).

## Materials and Methods

### Experimental Animals

Experiments were performed on 12 adult (P120) and 12 elderly (P1000) Mongolian gerbils (*Meriones unguiculatus*). This outbred species is an ideal model system for both multisensory and developmental research (e.g., Vincent et al., 1980; Cheal, 1986; Budinger and Scheich, 2009). Gerbils have good vision (also for colors; Jacobs and Deegan, 1994), hearing (with a human-like pronounced sensitivity to low frequencies; Ryan, 1976), touch (Cabana et al., 1993), and olfaction (Vallejo et al., 2000). They show anatomical, physiological, and behavioral evidence for crossmodal interactions, even at the level of primary sensory cortices (e.g., Cahill et al., 1996; Ohl et al., 2000; Budinger and Scheich, 2009; Kobayasi et al., 2013). Gerbils are sexually mature 2–3 months after birth and live to an average of 3.5 years (e.g., Vincent et al., 1980; Cheal, 1986).

Experimental animals were all males and weighed 80–100 g. They were housed in standard laboratory cages in air-conditioned rooms (22°C, 12 h light-dark cycle); water and pellets were available *ad libitum*. Elderly animals were healthy and showed normal behavior, for example, startle responses to loud sounds and light flashes (see, e.g., Henschke et al., 2017), hindpaw withdrawal reflex, regular exploration of their home cage, and normal food and water intake. *In vivo* and *ex vivo* analyses were negative for cholesteatomas, glaucomas, skin lesions, external and internal tumors, edemas, inflammations and other severe diseases.

All experiments were performed according to the NIH Guide for the Care and Use of Laboratory animals (2011) and the Directive of the European Communities Parliament and Council on the protection of animals used for scientific purposes (2010/63/EU) and were approved by the animal care committee of Sachsen-Anhalt, Germany (42502-2-1324 LIN).

### Neuroanatomical Tracer Injections

For tracer injections, nine adult and nine elderly gerbils were used: three animals per age group and injection site into A1, S1, and V1. Animals were anesthetized with ketamine (10 mg/100 g body weight, ip) and xylazine (0.5 mg/100 g body weight, ip). The cranial skin was incised, the skull was exposed by a displacement of the skin and muscles, and a small hole was drilled into the skull. For craniotomies and tracer injections, which were always performed on the left side, we used the following stereotaxic coordinates derived from the gerbil brain atlas (Radtke-Schuller et al., 2016): A1: 2.8 mm rostral to lambda/6.5 mm lateral/1.5 mm deep; S1 hindlimb area (HL): 4.55/2.5/1 mm; V1: 0.7/3.2/1 mm. We injected 18 nl of the retrograde fluorescent tracer FG (hydroxystilbamidine; Fluorochrome, LLC, Denver, CO, USA; 10% solution in *Aqua dest*.) in 2 × 9 nl steps over 5 min into these areas via an oil hydraulic nanoliter delivery system (World Precision Instruments, Germany) and fine glass micropipettes (tip diameter 20 μm). After injections, craniotomies were sealed with bone wax (Ethicon, Johnson & Johnson, Germany), the surgical openings were treated with an anti-inflammatory ointment (Volon A; Dermapharm GmbH, Germany), and the skin was closed with tissue adhesive (Histoacryl; B/Braun, Germany). Thereafter, animals were allowed to recover and survive for 3 days.

### Histological Processing

Animals were deeply anesthetized with ketamine (20 mg/100 g body weight, ip) and xylazine (1 mg/100 g body weight, ip) and perfused transcardially with 20 ml of 0.1 M phosphate-buffered saline (PBS, pH 7.4) followed by 200 ml of 4% paraformaldehyde. The brains were removed, postfixed overnight in 4% paraformaldehyde at 4°C, and then cryoprotected by soaking them in 30% sucrose in PBS for 48 h. Brains were cut on a cryostat (Leica CM 1950, Germany) into 50 μm thick frontal sections. Every first and second section was directly mounted on gelatine-coated glass slides for pure visual inspection of FG labeling, every third section was collected in PBS (free-floating) and counterstained either against cleaved (activated) CASP3 (rabbit, 1:500, #Asp175, Cell Signaling, Danvers, MA, USA), GAP43 (rabbit, 1:500, #ab16053, Abcam, United Kingdom), or PV (mouse, 1:4000, #235, SWANT Switzerland) overnight at 4°C. After washing and blocking, sections were incubated with the respective secondary Cy3-labeled antibodies (1:500, anti-rabbit #111-165-047, anti-mouse #115-165-166, Dianova, Germany) for 2 h. Finally, sections were washed again, mounted on gelatin-coated slides, and coverslipped with MOWIOL (Fluka, Germany).

In addition, six brains of non-injected animals (three per age group) were used in order to investigate possible effects of aging on general cyto- or myeloarchitectural patterns of the sensory cortex and thalamus. To this end, 50 μm thick frontal sections were alternatively stained for Nissl using cresyl violet and against myelin using an antibody against the Myelin Basic Protein (MBP). For the myelin stain, sections were first incubated in the MBP antibody solution (rabbit, 1:100, #ab40390, Abcam; 0.1% Triton; overnight) followed by the secondary antibody solution (1:200, biotinylated anti-rabbit, #111-065-144, Dianova; 2 h). The antibody reaction was visualized using the avidin-biotin-perioxidase method (ABC kit, Vector Laboratories, Burlingame, CA, USA) with diamino-benzidine as the chromogen. Sections were then mounted on gelatin-coated slides, and coverslipped with Merckoglas (Merck, Germany).

### Data Analysis

All sections were thoroughly examined under a combined brightfield and fluorescent microscope (Leica DMRX, Germany) with the appropriate filter sets. FG has an excitation maximum of 323 nm and an emission maximum of around 540 nm (depending on pH, golden fluorescent, Leica filter set A) and Cy3 of 550 nm and 570 nm (red fluorescent, Leica filter set N2.1). Digital images were taken with a Nikon D7000 microscope-mounted camera. Illustrations were arranged, labeled, and slightly adjusted in brightness and contrast using the Adobe Photoshop software (v. 13.0.6 for Windows).

Cortical areas and subcortical structures were identified on the basis of their architecture and relative location using information derived from our previous studies (e.g., Henschke et al., 2015, 2017) and the stereotaxic gerbil brain atlas (Radtke-Schuller et al., 2016).

The extent of each injection site in terms of its width (W, measured parallel to the cortical layers) and length (L, measured across the injected layers) was evaluated, using the Line Measure Tool of the Neurolucida software (MicroBrightField, Williston, VT, USA), in the sections covering the center of each injection site (Table 1). The volume (*V*_i_) of injection sites was calculated considering their roughly cylindrical size using *V*_i_ = (π/4) × W^2^ × L.

**Table 1:** Case numbers of experimental animals, cortical areas and layers of Fluorogold (FG) injections, extent and size of tracer injection sites.

Case	Area of tracer injection	Injection layers-center	Injection layers-extent	Injection size-width^*1^ [μm]	Injection size-length^*2^ [μm]	Volumes injection sites [mm^3^]
P120–1	A1	all	all	721	1050	1.71
P120–2	A1	IV–V	all	622	1000	1.22
P120–3	A1	III–V	all	831	1113	2.41
P120–4	S1	all	all	708	912	1.44
P120–5	S1	IV	I–V	524	715	0.62
P120–6	S1	IV–V	all	428	812	0.47
P120–7	V1	all	all	661	973	1.34
P120–8	V1	all	all	634	901	1.14
P120–9	V1	IV–V	II–VI	541	884	0.81
P1000–1	A1	III–V	all	523	1016	0.87
P1000–2	A1	all	all	922	826	2.21
P1000–3	A1	all	all	600	1012	1.14
P1000–4	S1	IV–V	I–V	700	710	1.09
P1000–5	S1	V	III–VI	669	612	0.86
P1000–6	S1	IV	I–V	638	805	1.03
P1000–7	V1	IV–V	all	467	827	0.57
P1000–8	V1	all	all	736	851	1.45
P1000–9	V1	IV–V	all	525	804	0.70

*1 measured parallel to layers, *2 measured across injected layers.

The number of retrogradely labeled neurons (i.e., neurons, which clearly contained the tracer) in the brain structures of interest was counted in two thirds of all sections of each experimental animal (third section was used for immunohistochemistry; see above), summing up to several thousand neurons per animal (Figure 3). For this study, we focused on sensory structures of the cortex and thalamus; namely, the primary and secondary sensory cortices and the sensory thalamic nuclei (for definition, see e.g., Groenewegen and Witter, 2004; Jones, 2007; Kirkcaldie, 2012; Table 2).

**Figure 1:**
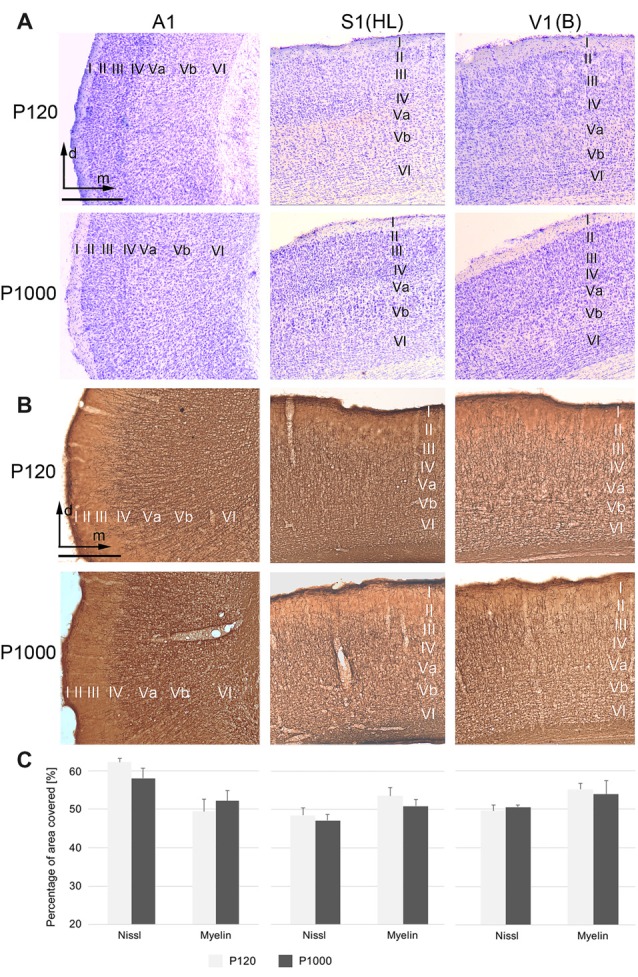
The general cyto- and myeloarchitectural patterns of the primary sensory cortices do not change over age.** (A,B)** Frontal sections showing the cytoarchitecture (Nissl; **A**) and myeloarchitecture (MBP; **B**) of the primary auditory (A1), somatosensory (S1, hindlimb area HL), and visual cortex (V1, binocular subfield B) cortex in adult (P120) and elderly animals (P1000). Scale bars: 500 μm. I–VI, cortical layers I–VI; d, dorsal; m, medial. **(C)** Percental area of A1 (right), S1 (middle), and V1 (left) covered by neuronal elements, i.e., by neuronal somata (Nissl) or myelinated fibers (myelin MBP). Values are percentages ±1 SEM, *n* = 9 sections per group.

**Figure 2:**
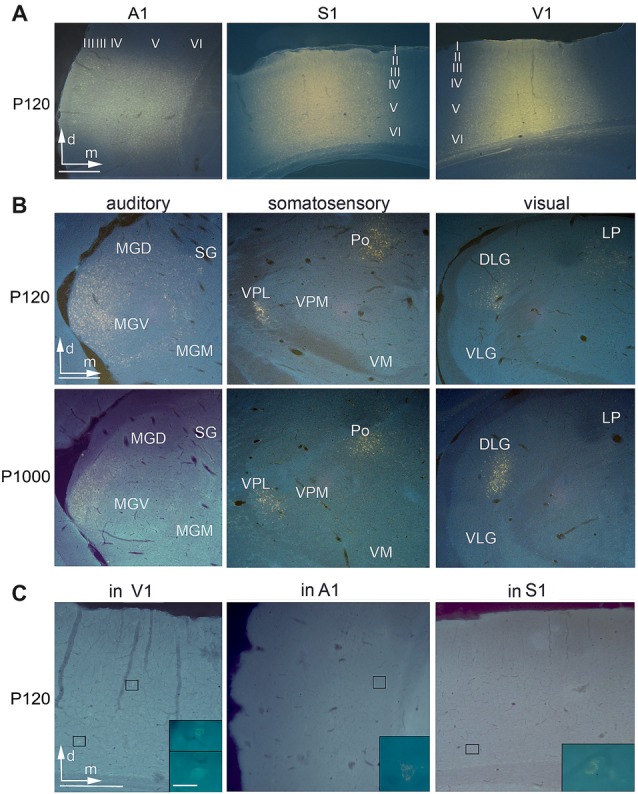
Anatomical tracer injections into the primary sensory cortices reveal multisensory connections at all ages.** (A)** Frontal sections showing injection sites of Fluorogold (FG) into A1, S1, and V1 at P120. **(B)** Retrogradely labeled cell bodies in auditory (MGD/M/V, dorsal/medial/ventral part of the medial geniculate body (MGB); SG, suprageniculate thalamic nucleus), somatosensory (Po, posterior; VM, ventromedial; VPL/M, ventral posterolateral/posteromedial thalamic nucleus), and visual (D/VLG, dorsal/ventral lateral geniculate nucleus; LP, lateral posterior thalamic nucleus) thalamic nuclei following tracer injections into A1, S1, and V1 at P120 and P1000. **(C)** Retrogradely labeled cell bodies in V1 after injection into A1, in A1 after injection into S1, and in S1 after injection into V1; always at P120. Scale bars 500 μm and 20 μm (insets).

**Figure 3:**
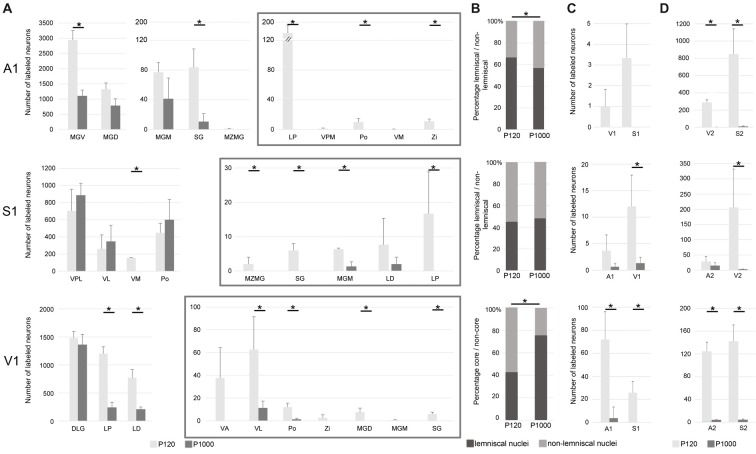
The number of most sensory thalamocortical and intracortical connections of A1, S1, and V1 decreases during aging.** (A)** Mean number ± 1 SEM of retrogradely labeled neurons in the sensory thalamic nuclei following tracer injections into A1, S1, and V1 listed for P120 and P1000. Non-matched (i.e., crossmodal) thalamocortical connections within each given modality are enframed (gray boxes). **(B)** Mean percentage of retrogradely labeled neurons in lemniscal (core) and non-lemniscal (non-core) thalamic nuclei on all sensory matched thalamic connections listed for P120 and P1000. **(C,D)** Mean number ± 1 SEM of retrogradely labeled somata in primary **(C)** and secondary **(D)** sensory cortices following tracer injections into A1, S1, and V1 listed for P120 and P1000. Stars indicate significant changes between experimental ages (always *p = 0.033, Kolmogorov-Smirnov (KS) test, *n* = 3 per age and injection site). Note the different scaling of the x-axes for better visualization of the values. Abbreviations: A1/2, primary/secondary auditory cortex; D/VLG, dorsal/ventral lateral geniculate nucleus; LD, laterodorsal thalamic nucleus; LP, lateral posterior thalamic nucleus; MGD/M/V, dorsal/medial/ventral part of the MGB; MZMG, marginal zone of the MGB; Po, posterior thalamic nucleus; S1/S2, primary/secondary somatosensory cortex; SG, suprageniculate thalamic nucleus; V1/2, primary/secondary visual cortex; VA, ventral anterior thalamic nucleus; VL, ventrolateral thalamic nucleus; VM, ventromedial thalamic nucleus; VPL/M, ventral posterolateral/posteromedial thalamic nucleus; Zi, zona incerta.

**Table 2:** Sensory thalamic nuclei containing retrogradely labeled somata after injections of FG into the gerbil’s A1, S1, and V1.

Auditory	*Lemniscal*	Medial geniculate body (MGB): ventral part (MGV)
	*Non-lemniscal*	MGB: dorsal part (MGD), medial part (MGM), marginal zone (MZMG), suprageniculate thalamic nucleus (SG)
Somatosensory	*Lemniscal*	Ventral posterolateral (VPL), ventral posteromedial (VPM) thalamic nucleus
	*Non-lemniscal*	Posterior (Po), ventral anterior (VA), ventromedial (VM), ventrolateral (VL, somatomotor) thalamic nucleus, zona incerta (Zi)
Visual	*Core*	Dorsal lateral geniculate nucleus (DLG)
	*Non-core*	Laterodorsal (LD), lateral posterior (LP), posterior limitans (PLi) thalamic nucleus

*Listed is their main modality and belonging to the lemniscal (core) or non-lemniscal (non-core) sensory pathways (e.g., Groenewegen and Witter, 2004; Jones, 2007)*.

For the analysis of Nissl and myelin staining intensities (Figure 1C), microscopic images of regions of interest were taken as described above, with 10× magnification and 15 ms exposure time and a constant brightfield illumination. RGB images were converted non-weighted into 8-bit gray-scale images and the background was subtracted using gray values of unstained cortical layer I (Image J, v. 1.43r, NIH, Bethesda, MD, USA). Then, gray-scale images were theresholded all in the same way and converted into bitmap images (black-white). Finally, the percental area covered by neuronal elements (i.e., either by cell bodies or by myelinated fibers) on the total area of the region of interest (i.e., A1, S1, V1) was measured in three consecutive sections in the three non-injected animals per age group using the Measurement Tool of Image J.

For the analysis of GAP43 and PV staining intensities (Figures 5, 6), microscopic images of regions of interest were taken with 10× magnification and 500 ms exposure time. Then, RGB images were converted non-weighted into 8-bit gray-scale images and mean gray values were determined in three consecutive sections covering the region of interest in three experimental animals per age group using the Measurement Tool of ImageJ.

**Figure 4:**
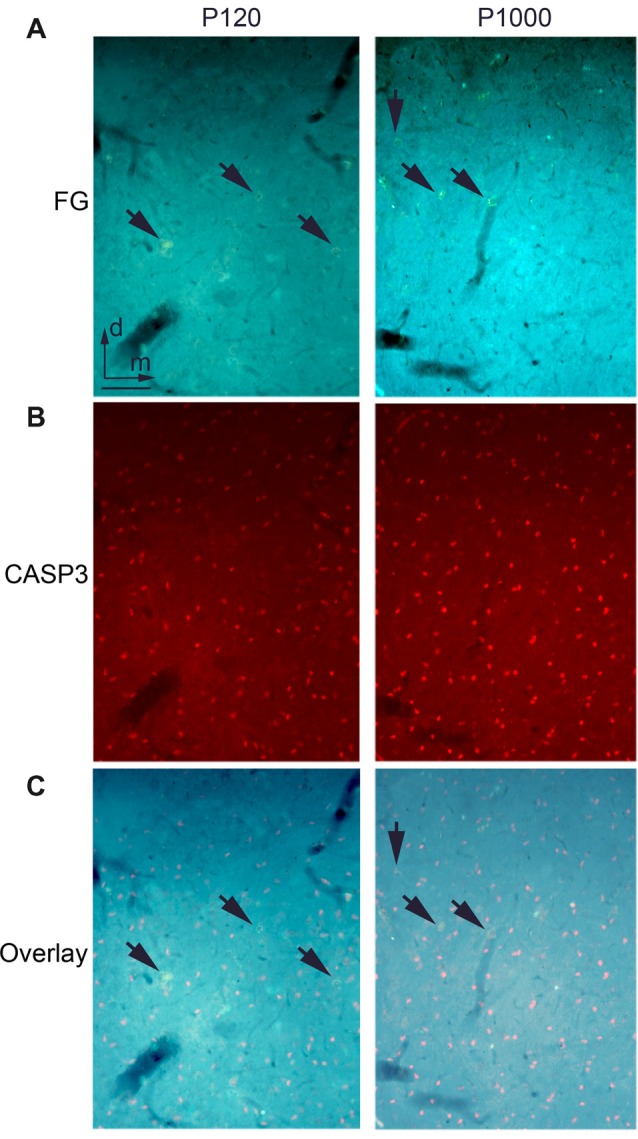
There is no ongoing apoptosis of projection neurons in elderly animals.** (A)** Frontal section through the auditory thalamus (dorsal part of the medial geniculate body, MGD) at P120 (left) and P1000 (right) showing retrogradely labeled neurons (golden, arrows) after an injection of FG into A1. **(B)** Same section showing apoptotic Cysteinyl-aspartate Specific Protease 3 positive (CASP3+) neurons (red). **(C)** Overlay of **(A,B)**, FG+ neurons appear golden (arrows), CASP3+ neurons appear pink. Note that there are no double-labeled neurons. Scale bar 25 μm.

**Figure 5:**
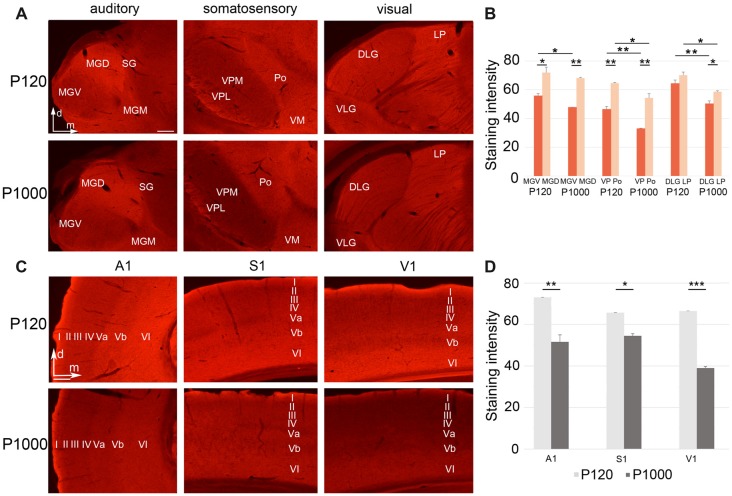
Growth associated protein 43 (GAP43) levels in thalamic nuclei and primary sensory cortices generally decrease with age.** (A,C)** Frontal sections showing GAP43 labeling in the auditory, somatosensory, and visual thalamic nuclei **(A)** and primary sensory cortices **(C)** at P120 and P1000. Scale bars 200 μm. **(B,D)** Average staining intensity of GAP43 in the sensory thalamus **(B)** and primary sensory cortices **(D)** at P120 and P1000. Note that the GAP43 levels are always higher in non-lemniscal nuclei (MGD, Po, LP) than in lemniscal nuclei (MGV, VPL/VPM, DLG). Staining intensities are mean gray values ± 1 SEM; stars indicate significant changes between structures and/or experimental ages (*p ≤ 0.05, **p ≤ 0.01, ***p ≤ 0.001, Student’s *t*-test; *n* = 9 sections per group). Abbreviations: A1, primary auditory cortex; D/VLG, dorsal/ventral lateral geniculate nucleus; LP, lateral posterior thalamic nucleus; MGD/M/V, dorsal/medial/ventral part of the MGB; Po, posterior thalamic nucleus; S1, primary somatosensory cortex; SG, suprageniculate thalamic nucleus; V1, primary visual cortex; VM, ventromedial thalamic nucleus; VPL/M, ventral posterolateral/posteromedial thalamic nucleus.

**Figure 6:**
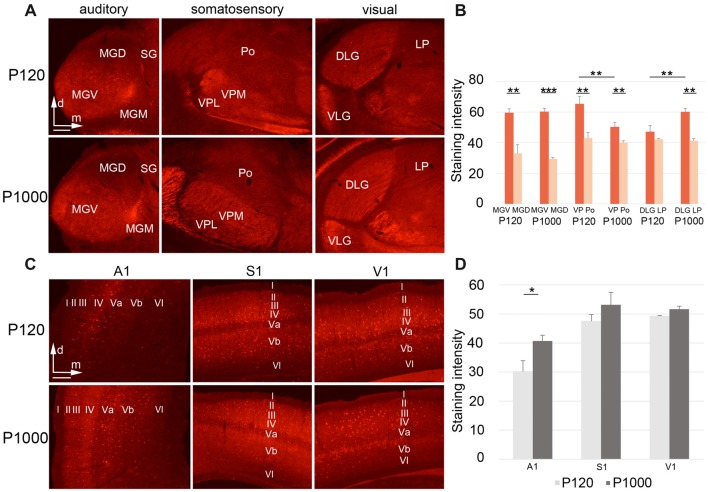
During aging, Parvalbumin (PV) levels change differently in the sensory thalamic nuclei and cortical areas.** (A,C)** Frontal sections showing PV labeling in the auditory, somatosensory, and visual thalamic nuclei **(A)** and primary sensory cortices **(C)** at P120 and P1000. Scale bars 200 μm. **(B,D)** Diagrams showing the staining intensities of PV in the sensory thalamus **(B)** and primary sensory cortices **(D)** at P120 and P1000. Note that the PV levels are always higher in lemniscal nuclei (MGV, VPL/VPM, DLG) than in non-lemniscal nuclei (MGD, Po, LP). Staining intensities are mean gray values ± 1 SEM; stars indicate significant changes between structures and/or experimental ages (*p ≤ 0.05, **p ≤ 0.01, ***p ≤ 0.001, Student’s *t*-test; *n* = 9 sections per group). Abbreviations: A1, primary auditory cortex; D/VLG, dorsal/ventral lateral geniculate nucleus; LP, lateral posterior thalamic nucleus; MGD/M/V, dorsal/medial/ventral part of the MGB; Po, posterior thalamic nucleus; S1, primary somatosensory cortex; SG, suprageniculate thalamic nucleus; V1, primary visual cortex; VM, ventromedial thalamic nucleus; VPL/M, ventral posterolateral/posteromedial thalamic nucleus.

All statistical analyses, including two tailed, unpaired Student’s *t*-test for normally distributed values, non-parametric Kolmogorov-Smirnov (KS) test for small sample sizes, and multivariate non-parametric Scheirer-Ray-Hare (SRH) test (Sachs, 2002), were performed using Microsoft Excel (v. 13 for Windows) and The Real Statistics software (Charles Zaiontz; real-statistics.com). Due to always *n* = 3 per group for the KS test, the minimal possible *p* for this test was *p = 0.033.

## Results

### The General Cyto- and Myeloarchitecture of the Sensory Cortex and Thalamus Do Not Change Over Age

The primary (“core”) auditory cortex A1, which is located in the temporal region of the gerbil brain, shows a koniocortical cytoarchitecture characterized by a particularly well-developed granular layer IV (Figure 1A, left row; see also, e.g., Budinger et al., 2000a; Radtke-Schuller et al., 2016). The supragranular layers (I–III) of A1 show a higher cell packing density than its infragranular layers (V–VI). A1 is heavily myelinated, most notably within the infragranular and granular layers (Figure 1B left row).

S1, located within the gerbil parietal cortex, is also koniocortical and densely myelinated (Figures 1A,B, middle row). S1 comprises several cytoarchitecturally and functionally discernible subfields. In the present study, we focused on the area for the representation of the hindlimbs (area HL) because previous studies demonstrated crossmodal connections preferentially for HL (Budinger et al., 2006; Henschke et al., 2015). Compared to the adjacent trunk (Tr) and forelimb (FL) areas, the HL area has a higher cell density in layer IV and a stronger myelination in layer V.

V1 forms a distinct koniocortical area within the occipital region of the gerbil brain, which can be divided into at least two anatomically and functionally different subfields, i.e., a monocular (V1M) and a binocular (V1B) subfield (Figures 1A,B right row). Both fields are koniocortical and densely myelinated; however, V1B has a slightly more cell-dense layer IV and fiber-dense infragranular layers.

During aging (here compared between P120 and P1000), we could not detect light-microscopic changes in the general cyto- or myeloarchitectural patterns of the sensory cortical areas (i.e., laminar patterns, orientation of fibers; Figures 1A,B) nor in the overall number of cell bodies or myelinated fibers (Figure 1C). In Figure 1C, the percental area covered by cell bodies (Nissl) or myelinated fibers on the total areas of A1, S1, and V1 is plotted for P120 and P1000; there is no significant change of this ratio over age (always *p* > 0.05, Student’s *t*-test). Whether there are changes, for example, in the thickness of myelin sheaths around axons, as suggested previously (e.g., Peters, 2009), can only be resolved using higher magnification microscopic techniques (e.g., electron microscopy, EM).

With regard to thalamic structures, we refrain from a detailed description of the gerbil auditory, somatosensory, and visual thalamus in the present work and refer to the specific literature on that topic (e.g., Budinger et al., 2000b; Mylius et al., 2013; Saldeitis et al., 2014; Henschke et al., 2015, 2017; Radtke-Schuller et al., 2016). We do emphasize however, that the overall cyto- and myeloarchitecture of the sensory thalamic nuclei did not show significant changes over age.

### Injection Sites Were Cylindrical and Covered Most of the Cortical Layers

In this study, only experimental cases were used where injections could be verified to have been unequivocally administered into the aimed target areas (A1, S1/HL, V1; for architectonic criteria see above) and where the tracers did not spread into the white matter or adjacent cortical areas. Generally, the laminar extent, size, and volume of the injection sites were similar across the experimental cases and ages (Table 1).

Injection sites in A1 were roughly cylindrical and had similar average volumes at P120 (1.78 ± 0.49 mm^3^) and P1000 (1.41 ± 0.58 mm^3^; *p* = 0.320, KS test). Injections were always placed in the center of A1 and thus comprised neurons representing high (>1 kHz) and low frequencies (<1 kHz). According to the size of the injection sites and the resolution of the tonotopic gradient across A1, the injection sites covered a frequency-representation area of approximately 1.5–2.5 octaves (200 μm/octave > 1 kHz, 400 μm/octave <1 kHz; Scheich et al., 1993; Ohl et al., 2000). The injections were centered in the middle cortical layers (III–V), but spread to all cortical layers (Table 1, Figure 2A left).

Likewise, injection sites in S1/HL were roughly cylindrical with a similar average volume at P120 and P1000 (0.84 ± 0.43 mm^3^ and 0.99 ± 0.10 mm^3^; *p* = 0.320). Injections were placed into HL close to Tr. The centers of the injections were in layers IV–V, but the injections extended over all cortical layers (Table 1, Figure 2A, middle).

The cylindrical injection sites in V1 had an average volume of 1.09 ± 0.22 mm^3^ (P120) and 0.90 ± 0.39 mm^3^ (P1000; *p* = 0.320). Injection sites always included parts of V1M and V1B and were typically centered in layers IV–V but also extended into the other layers (Table 1, Figure 2A, right).

Multivariate SHR analysis across the two experimental ages (i.e., P120 and P1000) and three different locations of the injection sites (i.e., A1, S1, V1) revealed neither the experimental age (*p* = 0.566) nor the location of the injection site (*p* = 0.073) as significant factor.

### The Number of Crossmodal Thalamocortical Connections Decreases During Aging

Injections into the primary sensory cortices A1, S1, and V1 revealed a large number of readily identifiable retrogradely labeled somata in several ipsilateral sensory thalamic nuclei (Figures 2A,B). For analysis, we grouped these nuclei into matched and non-matched modalities and lemniscal (core) and non-lemniscal (non-core) categories (Table 2, Figures 3A,B).

Following A1 injections, the number of neurons in the lemniscal ventral division (MGV) and non-lemniscal dorsal (MGD) and medial division (MGM) of the auditory medial geniculate body (MGB) as well as in the auditory suprageniculate nucleus (SG) decreased from P120 to P1000 (*p = 0.033, KS test, for MGV and SG; Figure 3A). Other projections were completely eliminated, such as in the marginal zone of the MGB (MZMG). Also, the ratio of labeled neurons in lemniscal MGV vs. the other non-lemniscal nuclei (MGD, MGM, SG, MZMG) decreased significantly from P120 to P1000 (*p = 0.033, KS test; Figure 3B). Notably, in the medial portion of the MGV, we found just rarely FG-labeled neurons at P1000, indicating reduced high-frequency connections between MGV and A1 (Budinger et al., 2000b; Saldeitis et al., 2014) in elderly animals. At P120, there was also a considerable number of crossmodal projections (4.2 ± 0.2% of all thalamic projections) from non-matched thalamic nuclei like the somatosensory ventral posteromedial (VPM), posterior (Po), and ventromedial thalamic nucleus (VM), zona incerta (Zi), and visual lateral posterior thalamic nucleus (LP) to A1. At P1000, all of these non-matched connections to A1 were completely absent (*p = 0.033, KS test).

Contrary to the other primary sensory areas, following S1 injections, the number of labeled somata in most lemniscal (ventral posterolateral thalamic nucleus, VPL) and non-lemniscal (Po, ventrolateral thalamic nucleus, VL) somatosensory nuclei slightly, but not significantly (*p* > 0.05, KS test), increased from P120 to P1000 (Figure 3A). One exception was the VM, where this number significantly decreased (*p = 0.033). The ratio of labeled cells in lemniscal vs. non-lemniscal somatosensory nuclei did not change from P120 to P1000 (*p* = 0.976, KS test; Figure 3B). At P120, we also found many crossmodal projections (2.5 ± 0.7% of all thalamic projections) from auditory MGM, SG, MZMG, and visual LP and LD (laterodorsal thalamic nucleus) to S1. At P1000, this number considerably decreased (0.3 ± 0.1%; *p = 0.033, KS test) including a significant decrease for MGM (*p = 0.033, KS test) and a total loss of the projections from MZMG, SG, and LP.

Following injections into V1, the number of neurons in the main (“core”) visual thalamic nucleus DLG (dorsal lateral geniculate nucleus) slightly, but not significantly (*p* = 0.320, KS test), decreased from P120 to P1000; however, there was a strong decrease in the non-core nuclei LP and LD (*p = 0.033, KS test; Figure 3A). The ratio of labeled cells in core vs. non-core visual nuclei increased from P120 to P1000 (*p = 0.033, KS test; Figure 3B). Similar to A1 and S1, during aging, the number of crossmodal projections significantly decreased from 3.4 ± 0.9% to 0.6 ± 0.2% (*p = 0.033, KS test) of all thalamic projections including a total loss of projections from somatosensory VA and Zi and auditory MGD, MGM, and SG.

In summary, at adult age (P120), most thalamocortical connections to primary sensory cortices were matched connections but there were also many non-matched connections (on average 3.35 ± 0.83%). During aging, the number of matched connections decreased in A1 and V1, but remained largely unchanged in S1. The ratio of lemniscal (core) vs. non-lemniscal (non-core) inputs to A1 decreased, increased for V1, and remained unchanged for S1. Non-matched (i.e., crossmodal) thalamic inputs strongly decreased for all three primary sensory cortices (on average down to 0.30 ± 0.32%). Concerning this decrease of crossmodal connections, multivariate SHR analysis across the two experimental ages and three different locations of the injection sites revealed experimental age (***p = 5.91·10^−4^), but not the location of the injection site (*p* = 0.746), as a significant factor.

### The Number of Crossmodal Intracortical Connections Strongly Decreases Over Age

Injections into A1, S1, and V1 also revealed a large number of retrogradely labeled somata in other ipsilateral primary and secondary sensory cortices (Figures 2A,C, 3C,D). This was in particular the case at P120. At P1000, all of these connections were strongly reduced or completely vanished, namely by 99.7% in A1, 92.1% in S1, and 96.7% in V1 (*p = 0.033, KS test; Figure 3C for primary, Figure 3D for secondary cortices). Also, multivariate SHR analysis showed that the experimental age (**p = 0.016 for connections between primary sensory cortices; **p = 1.26·10^−3^ for connections between primary and secondary sensory cortices), but not the injection site (*p* = 0.112 and *p* = 0.747, respectively), was the significant factor for this decrease.

### There Is No Ongoing Apoptosis of Crossmodally Projecting Neurons During Aging

To test whether the decrease of crossmodal projections is due to a programmed cell death of the respective projection neurons, we performed immunohistochemical staining for activated CASP3, which is a marker for ongoing cell apoptosis (e.g., Nicholson et al., 1995; Roth et al., 2000; see also Henschke et al., 2017).

At P120, we found several CASP3 positive (+) neurons in the thalamic nuclei (Figure 4A) and cortical areas. This number increased during aging (P1000, Figure 4B); however, at none of these time points, were the CASP3+ neurons double-labeled with FG (Figure 4C). This indicates that none of the FG-labeled (matched and non-matched) sensory thalamic and cortical projection neurons underwent apoptosis during the experiments. This notion is also supported by the only slightly (non-significant) decrease of the overall number of neurons in the primary sensory areas (Figure 1C).

### Decreasing GAP43 Levels Indicate a Reduced Axonal Plasticity in Very Old Animals

To investigate whether the decrease of crossmodal projections is instead due to a reduced axonal plasticity and thus concurring retraction of axons, we performed immunohistochemistry for GAP43, which is a widely used marker for ongoing axonal reorganization processes (e.g., Benowitz and Routtenberg, 1997; Holahan, 2017; see also Henschke et al., 2017).

In the sensory thalamus, the antibody against GAP43 mainly labeled the neuropil (Figure 5A). At P120 and P1000, we found a significantly less intense labeling of GAP43 in the lemniscal thalamic nuclei (MGV, VPM/VPL, DLG) compared to the non-lemniscal nuclei (MGD, Po, LP; always **p ≤ 0.01 or *p ≤ 0.05, Student’s *t*-test; Figure 5B), except for DLG-LP at P120 (*p* = 0.065). Further, at P1000, we found significantly reduced levels of GAP43 in all thalamic nuclei compared to P120 (always **p ≤ 0.01 or *p ≤ 0.05, Student’s *t*-test, Figure 5B), except for MGD.

In the primary sensory cortices, the GAP43 antibody also labeled mainly the neuropil (particularly in infragranular layers Va and VI; Figure 5C). Like in the thalamus, the GAP43 immunoreactivity in the primary sensory cortices significantly decreased during aging (**p = 0.003 for A1, *p = 0.013 for S1, ***p = 3.84·10^−6^ for V1, Student’s *t*-test; Figure 5D).

In summary, axonal plasticity and outgrow within the sensory thalamus and cortex are strongly reduced in elderly animals (P1000) compared to adults (P120). Further, axonal branches may also be retracted (Chao et al., 1992; Aigner and Caroni, 1995) from the primary areas leading to a reduced uptake (and subsequent retrograde labeling) of FG (see above).

### Altered Parvalbumin Levels in the Sensory Thalamus and Cortex Indicate Changing Excitation and Inhibition Patterns Over Age

Finally, we tested whether histochemical correlates of inhibition and excitation in the sensory thalamus and cortex change during aging. Therefore, we performed immunohistochemistry for the calcium-binding protein PV, which is a marker for inhibitory GABAergic (gamma-aminobutyric acid; e.g., Celio and Heizmann, 1981) and fast firing inhibitory and excitatory neurons in the brain (e.g., Celio, 1990; Cruikshank et al., 2001).

In the sensory thalamus, the PV antibody was found predominantly in the neuropil and only rarely in neuronal somata (Figure 6A). At both experimental ages, highest PV levels were always detected in the lemniscal sensory thalamic nuclei (***p ≤ 0.001, **p ≤ 0.01, or *p ≤ 0.05, Student’s *t*-test, Figure 6B), except for DLG-LP at P120 (*p* = 0.236). Towards P1000, the staining intensity significantly decreased in the lemniscal somatosensory thalamus (VPM/VPL; **p = 0.028, Student’s *t*-test), increased in the visual core nucleus (DLG; **p = 0.029, Student’s *t*-test), but stayed rather constant in the auditory and all other non-lemniscal (non-core) thalamic nuclei (*p* ≥ 0.05, Student’s *t*-test; Figure 6B).

In the primary sensory cortices, the PV antibody labeled the neuropil (particularly of layers II–IV) and the somata of (nearly exclusively) non-pyramidal neurons (Figure 6C). In elderly animals, the PV levels slightly increased, which was significant only for A1 (*p = 0.039, Student’s *t*-test; Figure 6D). There were no PV+ neurons double-labeled with FG, which was as expected since PV-expressing neurons in the cortex and thalamus are generally inhibitory interneurons without long-range projections.

Together, these results suggest that there are age-related, region-specific alterations of local inhibition and excitation both within and between the individual sensory thalamic nuclei and cortical areas.

## Discussion

In this study, using a rodent model (Mongolian gerbil), we provide anatomical evidence that there are direct thalamocortical and corticocortical connections across the auditory, somatosensory, and visual modalities that can relay crossmodal information to the earliest level of cortical processing, namely the primary sensory cortices A1, S1, and V1. In adult animals (P120), these crossmodal connections are substantial (e.g., on average 3.4% of all sensory thalamic inputs, which correlates to several thousand projection neurons; Budinger et al., 2006; Henschke et al., 2015). In elderly animals, crossmodal connections of the primary sensory cortices drastically decrease in number and many specific connections vanish entirely. Our present data indicate that this decrease is not due to an ongoing programmed cell death (apoptosis) of the respective projection neurons but rather to axonal retractions. Moreover, we found that these changes with age are accompanied by changes of local inhibition and excitation in the sensory cortex and thalamus.

Functionally, crossmodal connections in general enable the information transfer across the sensory modalities and improve the sensory performance of the individuals (crossmodal facilitation effect; Welsh and Warren, 1986; Stein and Meredith, 1993), for example, by decreasing their RTs (e.g., humans: Gielen et al., 1983; Teder-Sälejärvi et al., 2002; Noesselt et al., 2010; animals: Sakata et al., 2004; Gleiss and Kayser, 2012). Crossmodal connections at the level of the primary sensory cortices provide the structural basis for very short-latency multisensory integration processing in these areas (Sperdin et al., 2009; Henschke et al., 2015). Accordingly, neuronal responses with short latencies (<50 ms) to non-matched sensory stimuli have been reported to occur in A1, S1, and V1 of many species (e.g., monkeys: Brosch et al., 2005; Lakatos et al., 2007; Kayser et al., 2008; Wang et al., 2008; rodents: Iurilli et al., 2012; Sieben et al., 2013; humans: Giard and Peronnet, 1999; Foxe et al., 2000; Cappe et al., 2010; Raij et al., 2010). In turn, this very short-latency multisensory processing improves the behavioral performance of individuals to crossmodal stimuli by even further decreasing their RTs.

Older adults usually exhibit longer RTs to crossmodal stimuli than young adults (for review, e.g., Mozolic et al., 2012; Freiherr et al., 2013; de Dieuleveult et al., 2017). From the results of our study in a rodent model, one may suggest that also elderly human adults have fewer crossmodal connections at the level of the primary sensory cortices than young adults. Actually, this has not been tested so far although non-invasive tractography techniques like diffusion tensor imaging (DTI) are readily available in humans. However, more general DTI studies on normal aging (e.g., Salat et al., 2005; Giorgio et al., 2010; Yang et al., 2016) and studies on specific age-related sensory losses like presbycusis (e.g., Lutz et al., 2007; Profant et al., 2014; Ma et al., 2016) and glaucoma (e.g., Boucard et al., 2016) consistently revealed reduced subcortical and cortical white matter tracts and a decreasing fractional anisotropy (i.e., directionality of myelinated fibers) in sensory pathways of elderly humans, which may be indicative for reduced uni- and crossmodal connections.

In contrast to the putatively reduced crossmodal connectivity, older adults benefit more from crossmodal vs. unimodal stimulation than younger adults in terms of RT improvement (for review, e.g., Mozolic et al., 2012; Freiherr et al., 2013; de Dieuleveult et al., 2017). A possible explanation is that in older adults, short-latency multisensory processing is shifted from primary sensory cortical areas towards other brain regions. Indeed, older participants of a magnetoencephalography (MEG) study using an audiovisual detection task showed higher responses to crossmodal stimuli in posterior parietal and medial prefrontal cortex regions than young participants (Diaconescu et al., 2013). However, the crossmodal interactions in this study occurred between 150 ms and 300 ms after stimulus onset. Thus, interactions with shorter latencies may be shifted towards modality-specific subcortical structures, which have been shown to contribute to multisensory processing as well (e.g., animals: Shore et al., 2000; Ryugo et al., 2003; humans: Noesselt et al., 2010; van den Brink et al., 2014). Recent functional neuroimaging studies have revealed two more general patterns of age-related changes in brain activity across a variety of cognitive functions. The first is a more bilateral pattern of frontal recruitment in older adults named HAROLD (hemispheric asymmetry reduction in older adults; for review, e.g., Cabeza, 2002). The second pattern is an age-related reduction in occipitotemporal activity coupled with an increase in frontal activity named PASA (posterior-anterior shift in aging; for review, e.g., Davis et al., 2008). To date, these patterns have not been tested for multisensory function.

Another explanation for the enhanced multisensory benefit in older adults may be found in the principle of inverse effectiveness, which states that the decreased effectiveness of the individual unisensory stimuli can give rise to increased multisensory neuronal interactions (for review, e.g., Stein and Stanford, 2008). Thus, if there are decreased sensory-specific and crossmodal inputs into the primary sensory cortices of older adults (as observed in this study with elderly gerbils), this may facilitate inverse effectiveness and result in enhanced multimodal responses and higher RT benefits in older individuals. Psychophysical studies using audiovisual integration tasks with stimuli of varying intensities have provided evidence in favor of this idea. In one study (Hairston et al., 2003), young participants with normal vision were able to localize unimodal visual and bimodal audiovisual targets equally well; however, when participants’ vision was artificially degraded, their localization abilities were significantly enhanced during audiovisual conditions relative to the performance on visual targets alone. Yet, there is also evidence in contrast to this. A study on young and old participants demonstrated that in audiovisual conditions with easy visual stimuli, the integration enhancement measure (RT benefit) for older adults was equivalent to that for young adults and in conditions with hard visual stimuli, integration enhancement for older adults was significantly lower than that for young adults (Tye-Murray et al., 2011). Future studies should be designed in order to test possible correlations between decreased stimulus-specific inputs and multisensory enhancements at the physiological and behavioral level in animals and humans.

Other hypotheses about differences in multisensory integration abilities between young and older individuals are based on evidence that older adults use a larger time interval over which they integrate crossmodal stimuli (time window of integration: Diederich et al., 2008; Bedard and Barnett-Cowan, 2016), that older adults use sensory baseline information, which usually distracts younger adults (increased noise at baseline: Mozolic et al., 2012), and that older adults change their initial emphasis on stimulus characteristics to a greater emphasis on learned associations (dynamic reweighting: Murray et al., 2016). Again, future studies need to determine how such effects could be brought about by mechanisms on a circuit level.

Aging has been shown to result in cortical atrophy, due to shrinkage of white matter (degeneration of myelin sheaths; e.g., monkey: Peters, 2009) and also gray matter (10% cell loss in the range from 20 years to 90 years, e.g., human: Pakkenberg et al., 2003). Although, in this study, we did not observe an overall change in the myelination patterns of the sensory cortex and thalamus at the light-microscopic level, ultrastructural demyelination processes are likely to occur; as previous studies have observed this on the EM level in mouse A1 and V1 (Tremblay et al., 2012). We found an increase in cell apoptosis in elderly animals compared to younger ones, but this cell death did not include the labeled thalamocortical and corticocortical projection neurons. Nonetheless, we cannot entirely exclude that there has been neuronal cell death in these pathways during aging, which was not apparent with our CASP3 staining at P1000.

In any case, the reduced activity of GAP43, which is a marker for axonal outgrowth and plasticity (e.g., Benowitz and Routtenberg, 1997; Holahan, 2017), indicates a “silencing” and successive retraction of axons (Chao et al., 1992; Aigner and Caroni, 1995) in the sensory thalamus and cortex of elderly animals. This, in turn, reduces the ability of neurons to take up retrograde tracers and thus explains their decreased retrograde labeling.

We also observed region-specific changes in the expression patterns of the calcium-binding protein PV in sensory thalamus and cortex during aging, which is indicative of alterations in the balance between excitation and inhibition within and between these areas (e.g., Filice et al., 2016). On the one hand, PV is a marker for GABAergic inhibitory neurons particularly in the cortex (e.g., Celio and Heizmann, 1981). On the other hand, it is also abundant in the axons and terminals of fast firing, excitatory neurons, for example, of the sensory thalamus (e.g., Celio, 1990; Cruikshank et al., 2001). In accordance with previous studies on auditory (Long Evans rat: Ouda et al., 2008; human: Bu et al., 2003) and somatosensory cortex (mouse: Karetko-Sysa et al., 2014; mouse, gerbil, and rat: Ahn et al., 2017) we found slightly increasing PV levels in the sensory cortices of elderly gerbils compared to adult ones. This can be interpreted either as a result of increasing inhibition within the intracortical network or increasing excitatory input from the thalamus during aging (for review, e.g., Cruikshank et al., 2001; Ouda et al., 2015). Since our own tracing data show a substantial decrease of thalamocortical connections during aging, we would argue in favor of an increasing intracortical inhibition, but this is at least in contrast to theories of reduced inhibition in auditory cortex as a reason for age-related hearing deficiencies (for review, e.g., Caspary et al., 2008; Engle and Recanzone, 2012; Ouda et al., 2015). However, immunohistological studies on the co-expression of the GABA catalyzing enzyme GAD67 and PV in macaque auditory midbrain and brainstem have shown that the proportion of PV+ neurons expressing GAD67 changes over lifespan (decreasing and increasing depending on structure; Gray et al., 2014). Thus, it is not clear whether PV marks GABAergic inhibitory neurons in the same way in adult and elderly individuals. This might also be true for other markers of GABAergic inhibitory interneurons like somatostatin (SOM) and vasoactive intestinal peptide (VIP). In a comprehensive study on aging rat A1 cortex, authors showed that the number of PV+ and SOM+ significantly decreased over age (between P150 and P800), whereas the number of VIP+ neurons stayed rather constant (Ouellet and de Villers-Sidani, 2014). Interestingly, even the overall number of GABA+ neurons showed a barely significant downward trend after P150. Hence, further studies are needed to disentangle the relationship of age-related calcium-binding and other protein expression, cortical (and subcortical) balance of excitation and inhibition, neuronal connectivity, and sensory performance.

## Conclusion

In the present anatomical study, we show that the adult primary sensory cortices A1, S1, and V1 have substantial thalamic and intracortical connections across the sensory modalities. During aging, most of these crossmodal connections disappear. Additionally, these age-related changes in connectivity are most likely due to a retraction of the projection neuron axonal branches rather than their ongoing apoptosis. As a cause or consequence of the changing connectivity patterns, the local balance of excitation and inhibition in the sensory cortex and thalamus is altered. In view of the available functional data, the loss and restructuring of crossmodal connections during aging suggests a shift of short-latency multisensory processing from primary towards other sensory brain areas in older individuals.

## Author Contributions

JUH and EB: conceptualization and writing—original draft. JUH: investigation. JUH, EB and FWO: writing—review and editing. EB and FWO: funding acquisition.

## Conflict of Interest Statement

The authors declare that the research was conducted in the absence of any commercial or financial relationships that could be construed as a potential conflict of interest.
